# Measuring the statistical validity of summary meta‐analysis and meta‐regression results for use in clinical practice

**DOI:** 10.1002/sim.7372

**Published:** 2017-06-15

**Authors:** Brian H. Willis, Richard D. Riley

**Affiliations:** ^1^ Institute of Applied Health Research University of Birmingham U.K.; ^2^ Research Institute for Primary Care and Health Sciences Keele University U.K.

**Keywords:** validity, meta-analysis, models, statistical, data interpretation, statistical, decision making

## Abstract

An important question for clinicians appraising a meta‐analysis is: are the findings likely to be valid in their own practice—does the reported effect accurately represent the effect that would occur in their own clinical population? To this end we advance the concept of statistical validity—where the parameter being estimated equals the corresponding parameter for a new independent study. Using a simple (‘leave‐one‐out’) cross‐validation technique, we demonstrate how we may test meta‐analysis estimates for statistical validity using a new validation statistic, *Vn*, and derive its distribution.

We compare this with the usual approach of investigating heterogeneity in meta‐analyses and demonstrate the link between statistical validity and homogeneity. Using a simulation study, the properties of *Vn* and the *Q* statistic are compared for univariate random effects meta‐analysis and a *tailored meta‐regression* model, where information from the setting (included as model covariates) is used to calibrate the summary estimate to the setting of application. Their properties are found to be similar when there are 50 studies or more, but for fewer studies *Vn* has greater power but a higher type 1 error rate than *Q*. The power and type 1 error rate of *Vn* are also shown to depend on the within‐study variance, between‐study variance, study sample size, and the number of studies in the meta‐analysis. Finally, we apply *Vn* to two published meta‐analyses and conclude that it usefully augments standard methods when deciding upon the likely validity of summary meta‐analysis estimates in clinical practice. © 2017 The Authors. *Statistics in Medicine* published by John Wiley & Sons Ltd.

## 1 Introduction

The capacity to aggregate multiple studies and provide a summary estimate for translation into practice was one of the motivations that drove the development of meta‐analysis. In this regard, it has achieved undoubted success; however, the blight of heterogeneity, which so often affects meta‐analyses, can potentially affect the applicability of results in individual clinical settings, such as individual practices, hospitals, regions, or even countries.

Although methods have been developed to quantify and ascertain the effects of heterogeneity, more recently, particularly in the field of predictive modeling, the focus has been on developing statistical approaches that increase the validity of meta‐analysis results when applied in new populations. When evaluating diagnostic and prognostic tests, Riley and colleagues 1 examine approaches to translate test accuracy meta‐analysis results to a new population, and propose cross‐validation and prediction intervals to evaluate calibration performance of each approach. Debray and colleagues provide a framework for the use of individual patient data (IPD) from multiple studies in prediction modeling using logistic regression, and demonstrate that different model intercepts may be needed in new settings to ensure good predictive performance 2. Similarly, Snell et al. use IPD from multiple countries to develop and validate a breast cancer prognostic model, and show that it calibrates far better in each country when the baseline hazard is recalibrated (or ‘tailored’) to each country's population 3. A common theme to all of these approaches is the use of *cross‐validation* 4 where the *k* primary studies in the meta‐analysis provide all the data used in the validation process. The cross‐validation process involves comparing the meta‐analysis estimate from *k −* 1 studies with that estimate from the omitted study; this is repeated *k* times, each time omitting a different study.

The need to examine and improve the validity of meta‐analysis results should not be confined to the prediction modeling field. Indeed, assessing whether meta‐analysis results translate into practice should be the concern of all reviewers and statisticians producing summary results from a body of evidence, whether it is for the purpose of diagnosis, treatment, prognosis, or otherwise.

With this in mind, in this article we propose a general method for assessing the statistical validity of meta‐analysis results when applied in clinical practice. The predominant question we aim to address is when should we apply a summary meta‐analysis estimate to an independent setting? Specifically, if μ_ma_ is the parameter for the true summary effect of interest as estimated by the meta‐analysis analysis model and μ_setting_ is the parameter for the true effect in an independent setting of interest, then does μ_ma_ = μ_setting_? When the two are equal, we propose that the summary estimate from the meta‐analysis model can be described as having *statistical validity*. However, if the two are not equal, then meta‐analysis results may need to be modified (or ‘tailored’) to the setting of interest in order to ensure statistical validity. We will also examine statistical validity in the context of heterogeneity considering some of the methods used to establish heterogeneity.

We develop this in the following sections. We describe the meta‐analysis and tailored meta‐regression models in section 2. In section 3, we develop a new validation statistic, *Vn*, and derive its associated distribution applied to meta‐analysis and meta‐regression models. We consider how statistical validity relates to heterogeneity and compare *Vn* with Cochran's *Q* statistic. The properties of *Vn* and *Q* are examined more closely in a simulation study in section 4, and then application is made to two case examples in section 5. In the discussion in section 6, we consider its use and shortcomings.

## 2 Meta‐analysis and mixed‐models

Supposing there are multiple studies that each evaluate a particular effect of interest (e.g. an intervention effect, the sensitivity of a test, or the performance of a prognostic model). Of interest is the summary (mean) effect across studies and, for the purposes of this paper, how to apply or tailor such summary meta‐analysis estimates to clinical practice. Given the potential variation in the true effects between primary studies, we develop methods from the view point of a random effects model.

(i) *Meta‐analysis model*

For the observed mean effect, *y*_*i*_, in a primary study, we use the following univariate random effects model to aggregate the primary studies in the meta‐analysis

(1) $$y_{i}=\mu +\delta_{i}+\varepsilon_{i}$$

where *μ* is the mean (summary) effect across the studies and the key parameter to be estimated, *δ*_*i*_ ~ *N*(0, *τ*^2^) is the study‐specific deviation from the overall mean effect with unexplained between‐study variance *τ*^2^, and *ε*_*i*_ ~ *N*(0, *v*_*i*_) is the sampling error with variance *v*_*i*_ assumed known for each study *i*. This will be used as the base model in the meta‐analysis, where the key result for translation to clinical practice is the summary effect estimate, $\hat{y}$.

(ii) *Tailored meta‐regression model*

Heterogeneity across settings may be explained by study‐level covariates, and such covariates may be important when applying summary meta‐analysis results in clinical practice. We consider the effects of covariates on the meta‐analysis by incorporating these within a meta‐regression model. When there are *p* − 1 covariates, we have

(2) $$y_{i}=X_{i}\beta +\delta_{i}+\varepsilon_{i}$$

where ***X***_*i*_ is the row vector with *p* elements (the first element is 1) associated with study *i*, **β** is the *p*‐vector of coefficients to be estimated (the first element being the intercept term), and *δ*_*i*_ and *ε*_*i*_ are defined as previously. This model allows us to obtain summary results ‘tailored’ for particular populations of interest, defined by their set of covariate values and estimated by $X_{i}{\hat{\beta }}_{\left(-i\right)}$ where ${\hat{\beta }}_{\left(-i\right)}$ denotes the estimate of **β** derived from (2) with the *ith* study omitted. Therefore, following estimation of model (2), the key result for translation to clinical practice is $X_{i}{\hat{\beta }}_{\left(-i\right)}$. The merits of using information from the setting of interest have been recently described by Willis and Hyde 5, 6 particularly in the case of diagnosis. Essentially, it helps tailor the summary meta‐analysis estimate to the setting for clinical application, to potentially improve its validity.

Models (1) and (2) can be estimated using standard techniques, such as methods of moments and restricted maximum likelihood (REML). All meta‐analysis and meta‐regression models in this article are conducted in R using the package *Metafor* 7.

## 3 Examining summary meta‐analysis estimates in clinical practice

### 3.1 Cross‐validation approach

To evaluate whether meta‐analysis results may be translated into practice requires the development of methods that allow the cross‐validation of the derived summary estimates in new independent settings. In prognostic modeling, Royston and colleagues proposed what they called an ‘internal–external cross‐validation’ procedure to establish the generality of a prognostic model developed across different studies 4. More recently, this method has been elaborated upon by Riley 1 and Debray 2. Essentially, the procedure bears similarity with the ‘Jack‐knife method’ 8 by omitting each primary study, in turn, from the meta‐analysis and deriving a summary estimate from the remaining studies, which is then compared with the observed estimate in the corresponding omitted study. When there are *k* independent studies, the procedure generates *k* different meta‐analysis estimates, and thus *k* different validations. As such, the primary studies selected for the meta‐analysis are themselves being used as the basis for independent validation.

### 3.2 Validation statistic, *Vn*

In the remainder of this article, we focus on developing a statistic to test the validity of summary meta‐analysis estimates for clinical practice, within the context of the aforementioned cross‐validation approach. Let *y*_*i*_ be the observed mean effect estimate of interest in the *ith* study, and ${\hat{y}}_{\left(-i\right)}$ be the summary meta‐analysis estimate (from either model (1) or model (2)) generated from using *k* − 1 studies with the *ith* study omitted. Therefore, following the cross‐validation exercise, we have a dataset containing *k* values of *y*_*i*_ and ${\hat{y}}_{\left(-i\right)}$.

In this context, we propose the validation statistic, *Vn*

(3) $$\mathrm{Vn}=\sum_{i=1}^{k}\frac{{\left(y_{i}-{\hat{y}}_{\left(-i\right)}\right)}^{2}}{\mathrm{var}\left(y_{i}\right)+\mathrm{var}\left({\hat{y}}_{\left(-i\right)}\right)}$$

where var(*y*_*i*_) is the variance of *y*_*i*_ and $\mathrm{var}\left({\hat{y}}_{\left(-i\right)}\right)$ is the variance of ${\hat{y}}_{\left(-i\right)}$.

Assuming *y*_*i*_ to be normally distributed, the *Vn* statistic may be used as an overall test of the null hypothesis that *μ*_(−*i*)_ = *μ*_*i*_ for all *i*, where *μ*_(−*i*)_ is the parameter (true predicted effect) that underlies the ${\hat{y}}_{\left(-i\right)}$ predicted by the meta‐analysis model, and *μ*_*i*_ is the parameter (true effect) in the omitted study *i*. By our definition, when the null hypothesis is true, the meta‐analysis/regression estimate is a statistically valid estimate for a new setting. Thus, if we define a *p* value <0.05 for *Vn* as significant, then a *p* < 0.05 implies that there is sufficient evidence to conclude the meta‐analysis/regression estimate is not statistically valid.

In section 3.3, the distribution for *Vn* is derived for meta‐analysis/regression models.

### 3.3 Distribution of *Vn* for meta‐analysis and meta‐regression summary estimates

Here, we give an outline of the derivation of the asymptotic distribution of *Vn* recognising that *Vn* is a quadratic form and using an approach described in previous studies 9, 10. A more detailed description of the derivation is given in the appendix 1.

Assuming a continuous outcome, let the *ith* study have observed mean effect *y*_*i*_ and variance ${=\sigma }_{i}^{2}/n_{i}$ where $\sigma_{i}^{2}$ is the variance of the patient‐level observations in each study with sample size *n*_*i*_. By writing the weights, $w_{i}^{*}=1/(\left(\sigma_{i}^{2}/n_{i}\right)+\mathrm{var}\left({\hat{y}}_{\left(-i\right)}\right)$, *Vn* may be written as

(4) $$\mathrm{Vn}=\sum_{i=1}^{k}w_{i}^{*}{\left(y_{i}-{\hat{y}}_{\left(-i\right)}\right)}^{2}$$

If we define the *k × k* matrix **A** appropriately (see appendix 1), the term inside the squared brackets may be written as **Ay** **,** where **y** is the *k‐*vector with elements (*y*_1_, *y*_2_, *y*_3_,  … , *y*_*k*_), and *Vn* may be written in the following matrix form

(5) $$\mathrm{Vn}=y^{T}A^{T}w^{*}\mathrm{Ay}$$

The diagonal matrix **w** ^*^ has diagonal elements $\left(w_{1}^{*},w_{2}^{*},w_{3}^{*},\ldots w_{k}^{*}\right)$, and in general, the diagonal elements of **A** are all 1. The advantage of writing *Vn* in this form is that the null hypothesis, *μ*_(−*i*)_ = *μ*_*i*_ for all *i*, is equivalent to **Aμ** = 0 **.**

By transforming **y** into a vector **z** of standard normal variables and applying the *spectral decomposition theorem*, it may be shown that for eigenvalues *λ*_*i*_ of **B** = **w**^−1/2^**A**^**T**^w^*^Aw^−1/2^ where **w** is the diagonal matrix with diagonal elements $w_{i}=n_{i}/\sigma_{i}^{2}$

(6) $$\mathrm{Vn}∼\sum_{i=1}^{k}\lambda_{i}\;\chi_{1}^{2}$$

Thus, *Vn* has a distribution which is a linear combination of *χ*^2^ variables of degree 1. This is an exact distribution if the $\sigma_{i}^{2}$ are all known. In practice, the $\sigma_{i}^{2}$ are estimated from the sample data, so it is an asymptotic distribution. Note that *Vn* has the same form of distribution for both the univariate random effects meta‐analysis and tailored meta‐regression, but both **A** and **B** differ between the two cases (see appendix 1).

### 3.4 Farebrother's algorithm to implement *Vn*

To apply *Vn*, the distribution specified in (6) needs to be known. The distribution for a linear combination of chi‐square variables has received considerable attention over the years. In general, there is no closed form to the distribution so that it has to be obtained numerically and a number of approaches have been described. Sattherwaite in 1946 described an approximate method based on the observation that when the non‐zero eigenvalues all equal one the distribution simplifies to single chi‐squared distribution with *k* degrees of freedom 11. This suggests that a single distribution with an ‘effective’ number of degrees of freedom may provide a suitable approximation. Other approaches include inverting the characteristic function (Davies 12, 13) and applying numerical integration to a weighted sum of chi‐squared variables (Fleiss 14).

Ruben made a notable development when he demonstrated that a linear combination of chi‐square variables could be written as an infinite series 15. Importantly, he also showed that the truncation error after *n* terms had an upper bound which was dependent on a chi‐squared distribution, the coefficients of the terms in the expansion, and *n*—all of which could be estimated accurately^15^. Thus, an estimate of the exact distribution may be obtained for the truncated series with *n* terms, such that *n* is set to make the truncation error arbitrarily small 9. Ruben's method is incorporated within Farebrother's algorithm 16 and it is this algorithm we use when estimating the distribution of *Vn*. The version of Farebrother's algorithm applied below is from the package *CompQuadForm* in R 10. The R source code used to estimate the distribution for *Vn* for a case example may be found in appendix 2.

### 3.5 Heterogeneity and statistical validity

The *Vn* statistic may be used as a test of the null hypothesis H_0_: *μ*_*i*_ = *μ*_(−*i*)_ for all *i*. For the base meta‐analysis model (1) if we define $w_{\left(-i\right)j}=1/\left(\left(\sigma_{j}^{2}/n_{j}\right)+\tau_{\left(-i\right)}^{2}\right)$ for *j ≠ i*, then the null hypothesis is equivalent to the following for each *i*

(7) $$\mu_{i}-\frac{\sum_{j\neq i}^{k}w_{\left(-i\right)j}\mu_{j}}{\sum_{j\neq i}^{k}w_{\left(-i\right)j}}=0$$

It can be seen from this that the null hypothesis is only satisfied when *μ*_*i*_ = *μ*_*j*_ for all *i* ≠ *j*, that is, when there is no heterogeneity. In short, we have the intuitive result that the base meta‐analysis model will provide a summary estimate which is statistically valid to all clinical settings only when the studies comprising the meta‐analysis are homogeneous.

Writing *Vn* in matrix form is useful when considering the null hypothesis. As stated earlier, the null hypothesis equates to **Aμ** = 0 where **μ** is the *k‐*vector of parameters; in other words, the above equations are equivalent to the *kernel* or *null space* of **A** on **μ** **.** Using Gauss‐Jordan elimination, **A** may be reduced to echelon form, and the above result follows readily.

Heterogeneity is likely to exist in most meta‐analyses, and thus, in general, individual clinical settings will have a true effect that differs from the mean (summary) effect; thus, one would expect *Vn* to lead to the null hypothesis being rejected in most applications of meta‐analysis.

In contrast, *Vn* is likely to be more useful when considering the case of tailored meta‐analysis results, as derived from the tailored meta‐regression model in (2) where covariates are included. Reducing **A** to echelon form (see Appendix 1 for definition of **A**), it follows that if *μ*_(−*i*)_ = *μ*_*i*_ for all *i* then for *p* − 1 covariates

(8) $$\mu_{i}=\sum_{j=1}^{p}\alpha_{\mathrm{ij}}\mu_{k-p+j}$$

The dimension of the kernel of **A**, otherwise known as the *nullity*, is *p*—this follows from the *rank‐nullity theorem*, because the rank(**A**) = *k* − *p*. Therefore, if *μ*_*k* − *p* + 1_ ,.., *μ*_*k*_ are all known, then this constrains *μ*_*i*_ to values in *p*‐space (equation (8)). The values of the coefficients *α*_*i*1_ ,  … *α*_*ip*_ depend on the within‐study variance, the between‐study variance, and the values of the covariates for each study; when the covariates are continuous, there are an infinite number of possible solutions. In summary, within the *k‐*dimensional space of all possible values of (*μ*_1_ , *μ*_2_,…, *μ*_*k*_), there is a *p‐*dimensional sub‐space where the (*μ*_1_ , *μ*_2_,…, *μ*_*k*_) are statistically valid. Because in most cases, we expect to find *μ*_*i*_ ≠ *μ*_*j*_, that is, the primary studies are heterogeneous, there is still the potential for the meta‐regression model to provide predicted summary estimates that are statistically valid.

When the covariates are discrete, the possible values of (*μ*_1_ , *μ*_2_,…, *μ*_*k*_) that are statistically valid share the same *p‐*dimensional sub‐space as continuous covariates. However, the number of possible values of (*μ*_1_ , *μ*_2_,…, *μ*_*k*_) is constrained by the number of levels, contrasting a continuous covariate, which may be thought of as having an infinite number of levels. Thus, for a meta‐regression model that includes only a single dichotomous covariate each of the *μ*_*i*_ ∈ (*μ*_1_, *μ*_2_,  … , *μ*_*k*_) can have only one of two values for them to be statistically valid. For example, if *μ*_1_ = 2.5 and *μ*_2_ = 3.8, then the other *μ*_*i*_ will be either 2.5 or 3.8. Although there is strict heterogeneity (not all the *μ*_*i*_ are equal), the data divide into two homogenous sub‐groups. Thus, similar to meta‐analysis, statistically valid estimates arise when the sub‐groups of studies are homogeneous. In essence, the process of adding covariates to a meta‐regression model in order to ‘explain’ heterogeneity is a one of identifying homogenous sub‐groups and this makes statistical valid estimates more likely. In the limit, when the covariates are continuous, this is equivalent to there being an infinite number of homogenous sub‐groups.

### 3.6 Comparison of the *Q* statistic with *Vn*

In meta‐analysis, Cochran's *Q* statistic 17 is classically used to identify heterogeneity 18. Specifically, *Q* is used to test the null hypothesis of homogeneity, namely, H_0_: *μ*_*i*_ = *μ*_*j*_ for all *i* ≠ *j* 18 or equivalently, H_0_: *τ*^2^ = 0 where *τ*^2^ is the between‐study variance.

For a meta‐regression model, the use of the *Q* statistic may be extended to detecting residual heterogeneity. In this instance, *Q* is used to test H_0_: $\tau_{r}^{2}=0$ where $\tau_{r}^{2}$ is the residual between‐study variance and this corresponds to identifying homogenous sub‐groups of studies. Thus, if a covariate has *m* levels, then for each level the sub‐group of studies corresponding to that level satisfy *μ*_*i*_ = *μ*_*j*_ for all *i* ≠ *j*.

Whether the *Vn* statistic is applied to a meta‐analysis or meta‐regression model, it is a test of the null hypothesis H_0_: *μ*_*i*_ = *μ*_(−*i*)_ for all *i*, which we defined as statistical validity. However, as we have deduced already, this is equivalent to testing H_0_: *τ*^2^ = 0 in the case of meta‐analysis and H_0_: $\tau_{r}^{2}=0$ in the case of meta‐regression. In essence, *Vn* and *Q* test the same hypotheses but from different standpoints, one from a direction of statistical validity of predictions, the other from homogeneity.

The key to our definition of statistical validity is the comparison of the parameter for the model estimate with that of an independent study. This derives from our goal of determining whether the model produces an estimate which accurately represents that seen in a new clinical population. Specifically, we would like to know how close the meta‐analysis/regression estimate of the effect is to the effect seen in an independent study. By incorporating a leave‐one‐out cross validation approach, the *Vn* statistic directly captures the out of sample prediction error.

In contrast, Cochran's *Q* statistic measures the deviation of the studies from the overall summary estimate or regression line. As all the studies are used to derive these summary measures, the *Q* statistic does not directly quantify how well these summary estimates generalise to independent settings.

In the next section, the statistical properties of *Vn* are studied and compared with Cochran's *Q* statistic.

## 4 A simulation study

In order to study the properties of *Vn* and compare them with the *Q* statistic, meta‐analyses and meta‐regression models were simulated for different values of the following parameters: *τ* ^2^ (between‐study variance), $\sigma_{i}^{2}$ (variance of patient‐level observations), *n* (sample size for each individual primary study), and *k* (number of primary studies in each meta‐analysis/meta‐regression). The following values were used: *τ* ^2^ [0, 0.05, 0.1, 0.25, and 0.5]; $\sigma_{i}^{2}$ [0.0001, 0.01, 0.1, and 1]; *n,* [50, 100, 250, 500, and 1000]; and *k* [5, 10, 25, and 50]. The $\sigma_{i}^{2}$ and *n* were varied between meta‐analyses/regressions but not between the primary studies within the meta‐analysis/regression to allow us to study the contributions of these separately.

In practice, *Vn* and *Q* are calculated using the asymptotic estimates ${\hat{\sigma }}_{i}^{2}$ and ${\hat{\tau }}^{2}$ for the parameters $\sigma_{i}^{2}$ and τ^2^, respectively. To simulate these for each primary study, *y*_*i*_ was calculated by taking the mean of the *n* simulated observations from $y_{m,i}\sim N\left(\mu_{i},\sigma_{i}^{2}\right)$ for *m = 1,..,n*. The ${\hat{\sigma }}_{i}^{2}$ was estimated as the variance of the observations *y*_*m* , *i*_ around *y*_*i*_, and ${\hat{\tau }}^{2}$ was estimated using *y*_*i*_ and ${\hat{\sigma }}_{i}^{2}\;$ to fit the meta‐analysis/regression models via the *Metafor* package 7. The models were fitted using REML.

When evaluating the type 1 errors of *Vn* and *Q* using a meta‐analysis model, we simulated a single *μ*_*k*_ ~ *N*(0, 1) and set *μ*_1_ = *μ*_2_ =  …  = *μ*_*k*_. The meta‐regression models were simulated using a single continuous covariate for each study *x*_*i*_ ~ *N*(0, 1). Hence, when investigating the type 1 error of the meta‐regression models, two values of *μ*_*i*_ ~ *N*(0, *τ*^2^) for *i = k* − 1, *k*, were simulated, and the remaining *k* − 2 values of *μ*_*i*_ were deduced as linear combinations of *μ*_*k* − 1_ and *μ*_*k*_ as according to (8).

To evaluate the power of *Vn* and *Q* the true effect (parameter) for each primary study, *μ*_*i*_ was simulated from a normal distribution according to *μ*_*i*_ ~ *N*(0, *τ*^2^) for *i = 1,..,k.* These were checked to ensure that **Aμ** ≠ 0 and rejected if otherwise.

For different combinations of (τ^2^, $\sigma_{i}^{2}$, *n, k*), the type I error rate and the power of *Vn* and *Q* were determined for a critical value of *p* = 0.05. The rate of type I error and the power were estimated based on 40 000 meta‐analysis/meta‐regression replications for each (τ^2^, $\sigma_{i}^{2}$, *n, k*) combination.

### 4.1 Type 1 error rates for Vn and Q

In Table 1 the rates of type 1 error for *Vn* and *Q* when applied to the meta‐analysis model in (1) are given. Here, there is no heterogeneity (τ^2^ = 0), that is, *μ*_*i*_ = *μ*_*j*_ for all *i* ≠ *j*. In general, the type 1 error rate is dependent on the average sample size, *n*, in the individual studies and the number of studies in the meta‐analysis, *k*. This reflects the estimates ${\hat{\sigma }}_{i}^{2}$ and ${\hat{\tau }}^{2}$ being less prone to sampling error as *n* and *k* increase, respectively.

**Table 1:** Rate of type 1 error for Vn and Q for meta‐analysis.

*n*	*k* = 5		*k* = 10		*k* = 25		*k* = 50	
	Vn	*Q*	Vn	*Q*	Vn	*Q*	Vn	*Q*
50	0.083	0.061	0.075	0.065	0.078	0.075	0.088	0.085
100	0.080	0.058	0.066	0.056	0.066	0.060	0.065	0.064
250	0.073	0.052	0.062	0.052	0.056	0.054	0.057	0.057
500	0.076	0.052	0.060	0.052	0.052	0.051	0.055	0.054
1000	0.072	0.051	0.059	0.049	0.052	0.050	0.050	0.051

Probabilities are derived from simulations based on 40 000 meta‐analysis replications with $\sigma_{i}^{2}$ fixed at 0.1.

*n* = individual study sample size; *k* = number of studies.

When there are fewer studies *k* < 50, and smaller sample sizes, *n*, the type 1 error rate of *Q* is closer to the threshold of 5% than *Vn*. The null distribution of *Q* applied to meta‐analysis is a *χ*^2^ distribution of degree *k* − 1 when $\sigma_{i}^{2}$ is known exactly 19, and for *n* = 1000, where sampling error of ${\hat{\sigma }}_{i}^{2}$ is minimised, the type 1 error rate for *Q* is consistently around the 5% mark across different numbers of studies.

Table 2 provides the type 1 error rates for *Vn* and *Q* when applied to the meta‐regression model in (2) where one continuous covariate was included. As in the meta‐analysis model, when compared with *Vn*, *Q* has type 1 error rates which are closer to the 5% threshold. When *n* = 50 and *k* = 5, *Vn* has a type 1 error rate as high as 9.3% compared 5.3% for *Q*. In general, for both statistics when the sample size of the individual studies is small (*n* = 50), the type 1 error rate increases with *k*.

**Table 2:** Rate of type 1 error for Vn and Q for meta‐regression (with one covariate).

*n*	*k* = 5		*k* = 10		*k* = 25		*k* = 50	
	Vn	*Q*	Vn	*Q*	Vn	*Q*	Vn	*Q*
50	0.093	0.055	0.084	0.064	0.079	0.071	0.085	0.085
100	0.090	0.053	0.073	0.055	0.065	0.061	0.066	0.065
250	0.085	0.054	0.067	0.051	0.057	0.053	0.057	0.055
500	0.087	0.051	0.069	0.051	0.058	0.053	0.053	0.051
1000	0.087	0.050	0.069	0.052	0.056	0.051	0.051	0.052

Probabilities are derived from simulations based on 40 000 meta‐analysis replications with $\sigma_{i}^{2}$ fixed at 0.1.

*n* = individual study sample size; *k* = number of studies.

### 4.2 Power of Vn and Q

**Figure 1:**
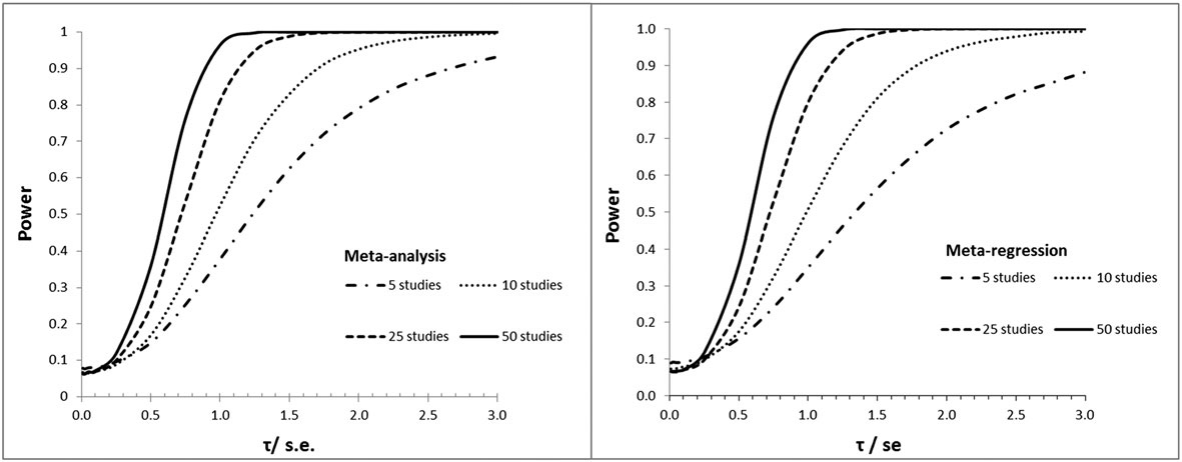
Power of *Vn*Meta‐analysis in left panel and meta‐regression in right. In both panels, we have *τ* varying, *σ* = 1, and *n* = 100.

In Figure 1 the power of *Vn* is plotted against *τ*/*se* (where $\mathrm{se}=\sigma_{i}/\sqrt{n}$) for models (1) and (2). In both the meta‐analysis and meta‐regression models, the power of *Vn* increases with increasing number of studies for a given *τ*/*se*. Thus, heterogeneity and large meta‐analyses increase the power of *Vn*.

Comparing the left and right panels, respectively, the power curves for *Vn* are shifted slightly to the right in the meta‐regression model when there are 10 studies or fewer. In short, the probability of rejecting statistical validity when it is known to be false for a given *τ*/*se* is more likely when *Vn* is applied to a meta‐analysis model than in a meta‐regression model with one covariate.

**Figure 2:**
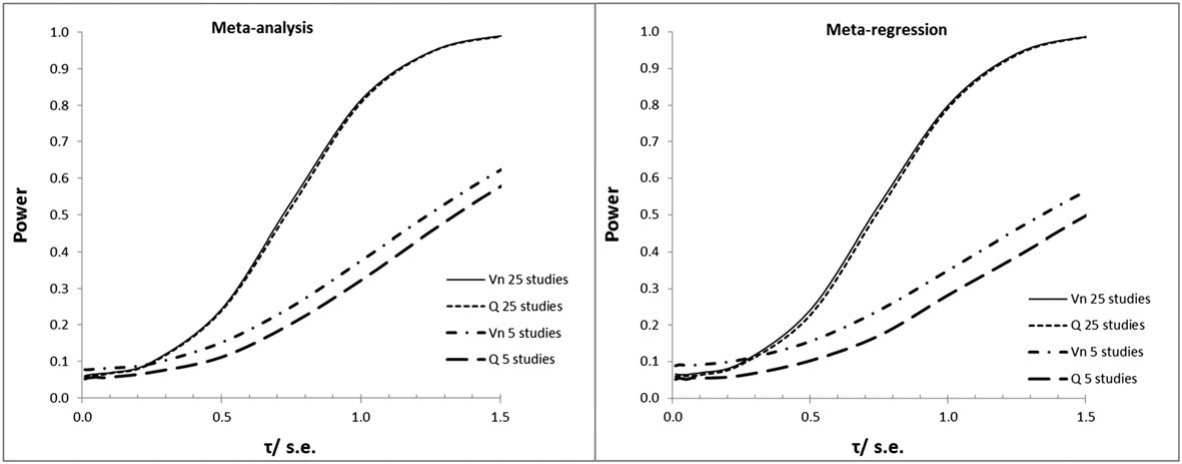
Comparison of power of *Vn* and *Q* for meta‐analysis and meta‐regression (with 1 covariate).

In Figure 2, the power of *Vn* and *Q* are compared for each of the models when there are 5 and 25 studies, respectively. When there are 5 studies, *Vn* has greater power than *Q* for a given *τ*/*se*. This difference is more pronounced in the meta‐regression model. When there are 25 studies in the analyses, the power curves for *Vn* and *Q* are closer, but *Vn* maintains greater power over *Q* for a wide range of *τ*/*se* *.* In short, *Vn* is more useful if we may assume heterogeneity and that estimates are more likely to be invalid.

Also of note is how the difference in type 1 error rates between the two statistics compares with the difference in power*.* When a meta‐analysis has 25 studies, the type 1 error rate of *Vn* is 0.001–0.006 higher than *Q* (this difference is 0.004–0.008 for meta‐regression). Although power varies with *τ*/*se*, for a similar‐sized meta‐analysis and *τ*/*se*< 1.5 the power of *Vn* is 0.002–0.014 higher than *Q* (difference in meta‐regression is 0.003–0.016). Essentially, the higher type 1 error rate of *Vn* compared with *Q* is compensated by a corresponding increase in power.

### 4.3 Interpretation

Central to proposing *Vn* is its use in testing the null hypothesis of statistical validity, but its interpretation has to involve weighing up the results in the context of other evidence. Before we interpret *Vn*, an important consideration is to decide whether the null hypothesis (statistical validity) is likely to be true. We know from 3.6 this is equivalent to deciding whether the studies are homogeneous and the cumulative evidence provided by such measures as the *Q* statistic 18, *I* ^*2*^ 18 and the width of prediction intervals 19 contribute to this decision. However, we should also be aware that none of these is without shortcomings when making such decisions 20, 21.

Depending on whether statistical validity seems likely, we then interpret the results of *Vn* in terms of the type 1 error rate or power (specifically the type 2 error rate). Here, the simulation study which evaluated these for both *Vn* and *Q* may be used to inform decision‐making when interpreting results.

If *Vn* is significant (*p* < 0.05), then either the meta‐analysis/regression estimates are invalid or there is a type 1 error. For *τ*/*se* > 3 and a 5% level of significance, the power of *Vn* is above 85% when there are 5 studies and above 99% when there are 10 studies. Thus, if model estimates are statistically invalid and *τ*/*se* is large enough, *Vn* will nearly always be significant. A type 1 error arises when the estimates are valid but *Vn* is significant. When there are few studies (*k =* 5) and the sample size is small (*n =* 50), *Vn* can have a type 1 error rate as high as 9% at a level of significance of 5%. Because validity is dependent on homogeneity (as we showed in 3.5), we may use the *Q* statistic in such instances as it maintains a type 1 error rate of around 5% even when *k* and *n* are small.

If *Vn* is not significant but statistical invalidity seems likely, then we should consider the type 2 error rate (1‐ power). The power of *Vn* is dependent on *k* and *τ*/*se*, and these are required for interpretation. We may estimate $\hat{\tau }$ directly from the meta‐analysis model and estimate the average standard error $\bar{se}$ of studies in the meta‐analysis based on that proposed by Higgins and Thompson 18, namely

$$\bar{se}={\left(\frac{\left(k-1\right)\sum w_{i}}{{\left(\sum w_{i}\right)}^{2}-\sum w_{i}^{2}}\right)}^{1/2}$$

where $w_{i}=n_{i}/\sigma_{i}^{2}$. Thus, any non‐significant results should be interpreted by weighing up whether they are consistent with the null hypothesis being true or there being a type 2 error given our estimate, $\hat{\tau }/\bar{se}$.

In order to gain some insight into the values of $\hat{\tau }/\bar{se}$ that may be expected, we screened the Cochrane database of Systematic Reviews for reviews published in September 2016. Including the two cases that follow, we found 16 reviews which provided summary 2 × 2 table data and had 5 or more primary studies (see Table 3). The median number of primary studies was 7 [lowest = 5, highest = 19] per review, and the median $\hat{\tau }/\bar{se}$ was 1.00 [lowest = 0, highest = 3.44]. Cochrane reviews tend to be of a higher quality than other reviews so they may not be representative—this may also account for the median primary study count being only 7.

**Table 3:** Values for $\hat{\tau }$ and $\bar{se}$ from a sample of meta‐analyses.

Study	*k*	Outcome	$\hat{\tau }$	$\bar{\mathrm{se}}$	$\hat{\tau }/\bar{\mathrm{se}}$
Fraquelli	5	Log RR	0.000	0.440	0.000
Martineau	7	Log OR	0.000	0.637	0.000
Clarke	9	Log OR	0.000	1.394	0.000
Wong	10	Log OR	0.000	1.087	0.000
Sheppard	7	Log RR	0.204	0.329	0.620
Greenough	7	Log RR	0.263	0.410	0.642
Prabhakar	6	Log RR	0.304	0.425	0.716
Sng	7	Log RR	0.475	0.484	0.980
Chin	9	Log RR	0.470	0.462	1.018
van Driel	6	Log OR	0.328	0.315	1.040
Kakkos	11	Log OR	0.906	0.782	1.158
Leeflang	7	Logit PPV	0.494	0.409	1.209
Wilkinson	6	Log RR	0.296	0.180	1.649
Bighelli	6	Log RR	0.557	0.292	1.911
Theron	19	Logit sens	1.069	0.471	2.271
Berkey	13	Log RR	0.560	0.163	3.444

RR = relative risk; OR = odds ratio; PPV = positive predictive value; sens = sensitivity.

For four studies, $\hat{\tau }$ = 0 and hence $\hat{\tau }/\bar{se}$ = 0 where both *Vn* and *Q* have low power. But in such instances, the power becomes less relevant because $\hat{\tau }$ = 0 suggests homogeneity. This reinforces the need to decide on whether statistical validity is likely to be true before interpreting the results.

When $\hat{\tau }/\bar{se}$ = 1, the power for *Vn* is 37.5% for *k* = 5 and is 52% for *k* = 10. This rises to 62% and 83%, respectively, when $\hat{\tau }/\bar{se}$ =1.5. There were four reviews in which $\hat{\tau }/\bar{se}$, and two of these had 6 studies. The lists of reviews are available in an online appendix.

We will now apply these principles to the following case examples.

## 5 Case examples

We now use two data sets to illustrate the use of *Vn* in testing the statistical validity of summary meta‐analysis results. In each case, we first fit the meta‐analysis model then the tailored meta‐regression model. Note all parameters were estimated from fitting the models using REML.

### 5.1 Berkey

The first dataset is from Berkey et al. 22 who reviewed the primary studies which evaluated the efficacy of the Bacillus Calmette–Guérin (BCG) vaccination in preventing tuberculosis (TB) 22. For both the meta‐analysis model and the tailored meta‐regression model, *Vn* is calculated as follows

$$\mathrm{Vn}=\sum_{i=1}^{13}\frac{{\left(\log{\left(\mathrm{RR}\right)}_{i}-{\hat{\log\left(\mathrm{RR}\right)}}_{\left(-i\right)}\right)}^{2}}{\mathrm{var}\left(\log{\left(\mathrm{RR}\right)}_{i}\right)+\mathrm{var}\left({\hat{\log\left(\mathrm{RR}\right)}}_{\left(-i\right)}\right)}$$

where ${\hat{\log\left(\mathrm{RR}\right)}}_{\left(-i\right)}$ is the summary estimate from the meta‐analysis/regression model fitted with the *ith* study omitted and log(*RR*)_*i*_ is the individual study estimate for study *i*. The individual study variance*, var(log(RR) = 1/tp + 1/cp – 1/(tp + tn) – 1/(cp + cn)* where *tp* and *tn* are the number of TB positive and negative patients in those who were vaccinated; *cp* and *cn* are the number of TB positive and negative patients in those who were not vaccinated. The $\mathrm{var}({\hat{\log\left(\mathrm{RR}\right)}}_{\left(-i\right)})$ is the model variance.

In Table 4 the individual study estimates for log(*RR*)_*i*_ are given alongside the corresponding meta‐analysis and tailored meta‐regression estimates for ${\hat{\log\left(\mathrm{RR}\right)}}_{\left(-i\right)}$. This shows directly how well the meta‐analysis and tailored meta‐regression estimate predicts that observed in the excluded study.

**Table 4:** Meta‐analysis and tailored meta‐regression estimates with study estimates using data from Berkey 22.

No.	Study	Year	Lat	Study estimate	MA estimate	TMR estimate
1	Vandiviere et al.	1973	19	−1.62 (−2.55, −0.70)	−0.66 (−1.01, −0.30)	−0.22 (−0.44, 0.00)
2	Ferguson & Simes	1949	55	−1.59 (−2.45, −0.72)	−0.65 (−1.01, −0.30)	−1.31 (−1.76, −0.85)
3	Hart & Sutherland	1977	52	−1.44 (−1.72, −1.16)	−0.63 (−0.97, −0.28)	−1.17 (−1.64, −0.70)
4	Rosenthal et al.	1961	42	−1.37 (−1.90, −0.84)	−0.65 (−1.01, −0.29)	−0.92 (−1.18, −0.65)
5	Rosenthal et al.	1960	42	−1.35 (−2.61, −0.08)	−0.69 (−1.05, −0.32)	−0.96 (−1.23, −0.69)
6	Aronson	1948	44	−0.89 (−2.01, 0.23)	−0.71 (−1.08, −0.33)	−1.04 (−1.33, −0.74)
7	Stein & Aronson	1953	44	−0.79 (−0.95, −0.62)	−0.71 (−1.10, −0.32)	−1.10 (−1.42, −0.79)
8	Coetzee & Berjak	1968	27	−0.47 (−0.94, 0.00)	−0.74 (−1.13, −0.36)	−0.55 (−0.81, −0.29)
9	Comstock et al.	1974	18	−0.34 (−0.56, −0.12)	−0.76 (−1.14, −0.37)	−0.27 (−0.64, 0.10)
10	Frimodt‐Miller et al.	1973	13	−0.22 (−0.66, 0.23)	−0.76 (−1.14, −0.39)	−0.11 (−0.54, 0.31)
11	Comstock et al.	1976	33	−0.02 (−0.54, 0.51)	−0.78 (−1.14, −0.41)	−0.75 (−0.93, −0.57)
12	TPT Madras	1980	13	0.01 (−0.11, 0.14)	−0.79 (−1.15, −0.44)	−0.22 (−0.68, 0.25)
13	Comstock & Webster	1969	33	0.45 (−0.98, 1.88)	−0.76 (−1.12, −0.40)	−0.73 (−0.94, −0.52)

The study estimate is the log(relative risk) for the individual study. The meta‐analysis (MA) estimate for a study is that derived from aggregating the remaining studies. The tailored meta‐regression (TMR) estimate for a study is derived from regressing the remaining studies with the covariate Lat but inserting the Lat value for the excluded study. For example, the MA estimate for study 1 is derived from aggregating studies 2–13. The TMR estimate for study 1 is derived from regressing studies 2–13 but inserting Lat = 19.

All estimates are for the log(RR) with 95% confidence intervals in brackets. Lat = latitude.

From Table 5, *Vn* (59.96; *p* < 0.0001) is significant, and as $\hat{\tau }/\bar{se}$ = 3.44, the power of *Vn* is likely to be close to 100% at a 5% level of significance. This strongly suggests the meta‐analysis estimate is unlikely to be valid in a new setting. The other statistics in Table 5 also suggest that there is heterogeneity which would be consistent with the estimate being invalid.

**Table 5:** Comparison of Vn with Q and I^2^ when applied to two case examples.

Cases	Outcome	95% PI	Vn	*p*‐Value	*Q*	*p*‐Value	*I* ^2^
1—MA	Log(RR)	(−1.87, +0.44)	59.96	<0.0001	152.23	<0.0001	92.2%
1—MR*	Log(RR)	(−1.67, −0.45)†	25.77	0.0037	30.73	0.0012	68.4%
2—MA	Logit(PPV)	(*−*1.84, +0.33)	16.76	0.0083	15.39	0.0175	59.75%
2—MR#	Logit(PPV)	(−1.19, −0.58)‡	6.04	0.484	4.86	0.433	0%

Case 1 (Berkey et al. [22]) and Case 2 (Leeflang et al. [23]). The results are given for the meta‐analysis (MA) and meta‐regression (MR) (with 1 covariate). *k* = the number of studies;

* Includes 1 covariate (the latitude)

# Includes 1 covariate (the logit(prevalence)); PPV—positive predictive value; RR—relative risk; 95% PI—95% prediction interval.

† Prediction interval estimated for a latitude of 45°.

‡ Prediction interval estimated for a prevalence of 10%.

When undertaking a meta‐regression, the reported efficacy in terms of the relative risk was associated with the latitude of the setting in which the study was conducted 22. Each fitted tailored meta‐regression equation with the *ith* study omitted took the form of:

(9) $$\hat{\log{\left(\mathrm{RR}\right)}_{\left(-i\right)}}={\hat{\alpha }}_{\left(-i\right)}+{\hat{\beta }}_{\left(-i\right)}\times {\text{latitude}}_{i}$$

where ${\hat{\alpha }}_{\left(-i\right)}$ and ${\hat{\beta }}_{\left(-i\right)}$ are estimated from fitting the model. It is of interest to know if the summary estimates from this tailored meta‐regression are valid in particular countries. Therefore, the cross‐validation approach is useful, with *Vn* used to test the calibration of the meta‐regression predicted effects and the actual study effects.

Although including the latitude as a covariate in the model has helped explain some of the heterogeneity (both *Q* and *I* ^*2*^ have decreased), *Vn* (25.77; *p* = 0.0037) remains significant. As noted above $\hat{\tau }/\bar{se}$ = 3.44, and for a tailored meta‐regression model, the power of *Vn* is still around 100%*—*this points strongly to the tailored meta‐regression estimate being invalid.

For the estimate to be statistically valid, we would need to interpret the significant result for *Vn* in the context of the probability of a type 1 error. Notwithstanding that Table 2 shows for a level of significance of 0.05 the true type 1 error rate of *Vn* can be higher than this, a *p* value of 0.0037 suggests a type 1 error is still very unlikely. This supports our judgment that the tailored meta‐regression estimates are likely to be statistically invalid.

### 5.2 Leeflang

The second dataset is derived from a Cochrane systematic review which appraised studies that had evaluated the Galactamannan assay for diagnosing invasive aspergillosis in immunocompromised patients 23. Here, we use the dataset with the threshold for a positive test result set at an optical density index (ODI) of 0.5. As in general, it is the probability of disease/non‐disease given the test result which is most useful to clinicians, the outcome of interest chosen in this example was the positive predictive value (PPV). Thus, the *Vn* statistic for both the meta‐analysis and meta‐regression model is calculated as follows

$$\mathrm{Vn}=\sum_{i=1}^{7}\frac{{\left(\text{logit}{\left(\mathrm{PPV}\right)}_{i}-{\hat{\text{logit}\left(\mathrm{PPV}\right)}}_{\left(-i\right)}\right)}^{2}}{\mathrm{var}\left(\log\mathrm{it}{\left(\mathrm{PPV}\right)}_{i}\right)+\mathrm{var}\left({\hat{\text{logit}\left(\mathrm{PPV}\right)}}_{\left(-i\right)}\right)}$$

The individual study variance *var[logit(PPV)*_*i*_ *] = 1/[(tp + fp)PPV(1‐PPV)]* where *tp* and *fp* are the number of true and false positive patients. The individual study estimates for log(*PPV*)_*i*_ with the corresponding meta‐analysis and tailored meta‐regression estimates for ${\hat{\log\left(\mathrm{PPV}\right)}}_{\left(-i\right)}$ are given in Table 6.

**Table 6:** Meta‐analysis and tailored meta‐regression estimates with study estimates using data from Leeflang 23

Author	Year	lgtprev	Study estimate	MA estimate	TMR estimate
Allan	2005	−4.82	−3.09 (−5.93, −0.26)#	−0.69 (−1.18, −0.20)	−2.65 (−4.06, −1.24)
Florent	2006	−2.56	−1.58 (−2.34, −0.82)	−0.59 (−1.02, −0.15)	−0.99 (−1.42, −0.56)
Kawazu	2004	−2.53	−0.74 (−1.46, −0.02)	−0.77 (−1.38, −0.15)	−1.25 (−1.68, −0.82)
Foy	2007	−2.21	−0.15 (−1.24, +0.94)	−0.84 (−1.38, −0.29)	−0.95 (−1.27, −0.63)
Yoo	2005	−2.10	−0.73 (−1.42, −0.05)	−0.77 (−1.39, −0.15)	−0.81 (−1.19, −0.43)
Weisser	2005	−1.95	−0.94 (−1.52, −0.36)	−0.72 (−1.34, −0.10)	−0.62 (−0.98, −0.26)
Suankratay	2006	−0.66	+0.21 (−0.52, +0.94)	−0.93 (−1.27, −0.59)	+0.26 (−1.20, +1.73)

The study estimate is the logit(PPV) for the individual study. The meta‐analysis (MA) estimate for a study is that derived from aggregating the remaining studies. The tailored meta‐regression (TMR) estimate for a study is derived from regressing the remaining studies with the covariate lgtprev but inserting the lgtprev value for the excluded study. For example, the MA estimate for study 1 is derived from aggregating studies 2–7. The TMR estimate for study 1 is derived from regressing studies 2–7 but inserting lgtprev = −4.82.

All estimates are for the logit(PPV) with 95% confidence intervals in brackets.

PPV = positive predictive value; lgtprev = logit(prevalence)

# Estimate includes continuity correction of 0.5.

From Table 5, for the meta‐analysis model, *Q* (15.39; *df* = 6; *p* = 0.0175), *I* ^*2*^ = 59.75% and the 95% prediction interval for the summary *logit* (*PPV*) is (−1.84, +0.33) (this is equivalent to a *PPV* of 14*–*58%)—these all suggest the studies are heterogeneous. As might be expected, *Vn (*16.76; *p* = 0.0083) is also significant which leads to the conclusion that any summary estimates are unlikely to be valid.

From Bayes' theorem, the *PPV* is known to depend on the disease prevalence. We implemented a tailored meta‐regression approach in which the prevalence of disease was assumed to be known for each primary study 5, 6, to study the effects of such information on the potential validity of any estimates. Thus, for the *Vn* statistic, each fitted tailored meta‐regression model took the form:

(10) $$\hat{\text{logit}{\left(\mathrm{PPV}\right)}_{\left(-i\right)}}={\hat{\alpha }}_{\left(-i\right)}+{\hat{\beta }}_{\left(-i\right)}\times \text{logit}{\left(\text{prevalence}\right)}_{i}$$

where ${\hat{\alpha }}_{\left(-i\right)}$ and ${\hat{\beta }}_{\left(-i\right)}$ are estimated from fitting the model with the *ith* study omitted.

In contrast to the first example, *Vn (6.04; p = 0.484)* is non‐significant. Should this be considered as consistent with the null hypothesis of statistical validity or is it a type 2 error? When there are few studies (*k =* 7), *Vn* has greater power and therefore a lower type 2 error rate than *Q.* From Table 5, $\hat{\tau }/\bar{se}$ = 1.21 and inspecting the power curves in Figure 2 we see the power is around 58% (a type 2 error rate of 42%) for a level of significance of 0.05. However, in this instance, the power for *Vn* will be much higher because *p* = 0.484. (A simulation study based on $\hat{\tau }/\bar{se}$ = 1.21 and an average sample size per study of 128 for the 7 studies demonstrates the power to be 90.0% giving a type 2 error rate of 10%). This would suggest that estimates are likely to be valid.

This is also supported by the other measures, as *I* ^2^ = 0%, and the prediction interval has narrowed to (*−*1.19, *−*0.58) equivalent to a *PPV* of 23*–*36% suggesting that the heterogeneity has been explained by the addition of the covariate to the model. Based on this evidence, it would be reasonable to implement a PPV estimate from this model in an independent setting and hence practice.

Both of these examples demonstrate why clinicians should not automatically assume summary meta‐analysis results are applicable to their population and that even when a summary estimate appears valid this should be judged in the context of the properties of the statistic and other evidence used to make that decision.

## 6 Discussion

One of the issues facing medical research in general is determining how well the research results translate into practice. To truly address this, we need a separate evaluation of the research in practice settings which are independent from the research settings. The terms validity or external validation are often reserved for when the research findings may be generalised or translated into clinical practice. With regard to meta‐analysis results, however, it is often impractical to conduct further independent studies to assess whether the aggregate estimates are valid. Judgments on validity are therefore generally based on an assessment of quality, in which a combination of qualitative and quantitative characteristics of the comprising studies is weighed up.

Yet the need for further studies is partly circumvented in meta‐analysis by making use of the existing studies in a similar way to the Jack‐knife method 7. Such ‘cross‐validation’ is not new and was implemented by Lachenbruch 24 and Stone 25 but has only recently gained some traction in meta‐analysis. However, to date, its main use in meta‐analysis has been in prediction modeling to evaluate predictive performance and for model recalibration 2, 3, 4.

To address this, we considered the concept of statistical validity in relation to estimates generated by meta‐analysis and meta‐regression models. In particular, we defined it as when the model parameter for the effect measure of interest equates to that in an independent setting. From this, and using the previously described cross‐validation procedure, we derived a statistic, *Vn*, and its asymptotic distribution to test the viability of statistical validity.

Homogeneity plays a central role and is integral to statistical validity when evaluating meta‐analysis or meta‐regression models with discrete categorical covariates. As part of the derivation of *Vn*, it was demonstrated that in meta‐analysis statistical validity follows only when the studies are homogenous. Furthermore, when meta‐analysis is extended to include discrete covariates in a tailored meta‐regression model, statistical valid estimates arise when the individual sub‐groups of studies are homogeneous, equivalent to the covariates being used to ‘explain’ the heterogeneity.

However, the more general case is when the *p* covariates are continuous in which case the set of statistical valid estimates spans a *p* + 1 dimensional sub‐space of the *k*‐space of parameters (*μ*_1_ , *μ*_2_,…, *μ*_*k*_). Here the individual parameters, *μ*_*i*_, may all have different values (by definition heterogeneity), but the model still provide statistically valid estimates.

Owing to the link between homogeneity and statistical validity, it reinforces the need to explore meta‐analyses/regressions for heterogeneity using standard methods 17, 18. As such, Cochran's *Q* statistic, a measure often used to identify heterogeneity, was compared with *Vn.* There are clear similarities in that they are both a ‘weighted sum of squares’ statistic. Because *Vn* is estimated using cross validation it directly measures the out of sample prediction error which is important to statistical validity and contrasts *Q*.

In terms of the type 1 error rate and power, they are similar when then are 50 or more studies. However, when there are fewer studies, *Vn* has greater power and *Q* has a lower type 1 error rate. Clearly defining the power and type 1 error rates is important as both these statistics are being used to test hypotheses. Furthermore, they provide a basis for recommendations on the use of *Vn* (and *Q*) when making decisions on the validity of meta‐analysis/regression estimates.

The power of *Vn* not only depends on the number of studies but also on τ/*se*, so there is a trade‐off between the level of heterogeneity and the average precision of the studies*.* Thus, when the meta‐analysis consists predominantly of high precision studies, *Vn* may detect differences between the model estimate and those observed which may be too small to be clinical relevant but are, nonetheless, statistically significant. However, our overview of Cochrane reviews showed this not to be a large problem.

As in previous studies 20, 26, similar shortcomings were demonstrated here with the *Q* statistic. This motivated the proposing of statistics, such as the *I* ^*2*^ statistic, that are aimed at measuring the extent of heterogeneity rather than its presence 27. However, this too is similarly affected by study precision and the number of studies in the meta‐analysis 20, 27, 28. Such drawbacks should inform the interpretation of these statistics and also suggest that they should not be used in isolation when evaluating heterogeneity or statistical validity.

Like many statistics, *Vn* provides little information when used in isolation; its usefulness depends upon the context in which it is applied. As statistical validity is intrinsically linked to homogeneity if homogeneity seems likely then a significant *Vn* result should be interpreted in terms of its potential type 1 error rate. In such an instance, the *Q* statistic, with its lower type 1 error rates, is likely to be the more informative of the two.

However, if heterogeneity is considered to be likely at the outset (as with many meta‐analyses), then *Vn's* greater power means that it could be more useful than the *Q* statistic particularly for *τ*/*se* < 1.5. In this context, reviewers may be able to use *Vn* as support for a recommendation which discourages the wider application of the meta‐analysis results.

But how should *Vn* be interpreted when the inclusion of covariates in meta‐regression analyses seems to ‘explain’ the between‐study heterogeneity? One of the risks in this instance is that legitimate exploratory analyses could lead to recommendations on validity. When the objective is to determine potential sources of variation and not make assertions on the ‘applicability’ of results, then the *Q* statistic and *I* ^*2*^ can be used to inform such analyses. Although in any event, a non‐significant *Vn* should be interpreted against the likelihood of a type 2 error, it should not be used to assert statistical validity in meta‐regression analyses when the covariates were identified as part of an exploratory phase. The risks of such data fishing or dredging are well documented 29 but are particularly apposite here where the results could be applied in clinical practice as a result. The best way to mitigate this risk is to pre‐specify the covariates that are likely to be causal before embarking upon any meta‐regression analyses. In this context, the assertion of statistical validity is more likely to be justified.

As an alternative to the hypothesis testing approach of the *Vn* statistic, Riley et al. propose using 95% prediction intervals to quantify the potential error in the predictions of *true* study values from applying meta‐analysis/regression models to new settings 1. This allows the magnitude of error to be examined, which may inform clinical relevance. Indeed, the issue of clinical relevance is pertinent to any methods used to assess validity. Altman and Royston allude to this when they express the importance of differentiating between statistical and clinical validity 30. In the latter case, biased estimates (which are statistically invalid) may be acceptable to clinicians under certain clinical conditions. However, there are potential limitations to the Riley et al. 1 approach, too. Prediction intervals are not universally accepted in the meta‐analysis field, and recent work suggests frequentist equations for the prediction interval have poor coverage 31. Furthermore, unlike the *Vn* statistic, the ‘error’ in the proposed prediction interval, as estimated using Riley's method 1, does not account for the variance of the meta‐analysis model's predicted summary estimate.

In this study, we confined our investigations to using study‐level data either as part of meta‐analyses or as covariates included in meta‐regression analyses. As IPD or patient‐level data become increasingly available, there is the potential to use IPD meta‐analyses to improve the summary estimates translated into practice. An important part of this process would be to evaluate their statistical validity and is worthy of future research.

In conclusion, for summary estimates from meta‐analysis to be useful in practice, they need to be statistically valid. As a direct measure of statistical validity, we have proposed the *Vn* statistic, and it is applicable whenever the meta‐analysis model (1) or the tailored meta‐regression model (2) is applied to combine effect estimates from multiple studies. It does have limitations as a hypothesis test, and these should be noted. However, as statistical validity relates to identifying homogenous sub‐groups of studies, these limitations may be partly circumvented by using it alongside other statistics such as the *Q* statistic. As such, *Vn* provides a useful summary of the likely statistical validity of results from meta‐analysis/regression models when applied to clinical practice.

## Supporting information

Data S1. Supporting info item

## Appendix 1 Distribution of *Vn*

### 1.1

(i) *Random effects meta‐analysis model (1)*

For the estimation of the univariate random effects meta‐analysis (1), we derive the asymptotic distribution of *Vn* by noting *Vn* is a quadratic form and applying results from linear algebra using an approach similar to Biggerstaff and Jackson 9 and Duchesne and Lafaye De Micheaux 10.

Let the *ith* study have observed mean effect *y*_*i*_ and variance ${=\sigma }_{i}^{2}/n_{i}$ where $\sigma_{i}^{2}$ is the variance of the patient‐level observations in each study with sample size *n*_*i*_. Let $\tau_{\left(-i\right)}^{2}$ be the between‐study variance when the *ith* study is excluded then we can then re‐write *Vn* as

$$\mathrm{Vn}=\sum_{i=1}^{k}w_{i}^{*}{\left(y_{i}-{\hat{y}}_{\left(-i\right)}\right)}^{2}=\sum_{i=1}^{k}w_{i}^{*}{\left[y_{i}-\frac{\sum_{j\neq i}^{k}w_{\left(-i\right)j}y_{j}}{\sum_{j\neq i}^{k}w_{\left(-i\right)j}}\right]}^{2}$$

where $w_{\left(-i\right)j}=1/\left(\left(\sigma_{j}^{2}/n_{j}\right)+\tau_{\left(-i\right)}^{2}\right)$ for *j ≠ i*, wi*=1/(σi2/ni+vary^−i=1/σi2/ni+1W−i and $W_{\left(-i\right)}=\sum_{j\neq i}^{k}w_{\left(-i\right)j.}$

This is more easily dealt with using a matrix formulation. Define *A*_*ij*_ by the following

(A1) Aij=1ifi=j−w−ijW−iifi≠j

Let **A** be the matrix with elements *A*_*ij*_, then **A** has rank = *k* − 1 (the entries in each row sum to zero). If **w** ^*****^ is the diagonal matrix whose diagonal elements are $\left(w_{1}^{*},w_{2}^{*},w_{3}^{*},\ldots w_{k}^{*}\right)$, and **y** is the *k‐*vector with elements (*y*_1_, *y*_2_, *y*_3_,  … , *y*_*k*_), then *Vn* may be written as the following

$$\mathrm{Vn}=y^{T}A^{T}w^{*}\mathrm{Ay}$$

Let **z** = **w**^1/2^**y**, where **w** is the diagonal matrix with diagonal elements $w_{i}=n_{i}/\sigma_{i}^{2}$ then we may write **B** = **w**^−1/2^**A**^**T**^**w**^*^**Aw**^−1/2^, hence

$$\mathrm{Vn}=z^{T}B\;z$$

**B** is a symmetrical matrix, and we may apply the *spectral decomposition theorem* to express *Vn* in terms of the eigenvalues and eigenvectors of **B** as previously reported 9, 10, namely

$$\mathrm{Vn}=\sum_{i=1}^{k}\lambda_{i}\;{\left(v_{i}^{T}z\right)}^{2}$$

where *λ*_*i*_ is the *ith* eigenvalue, **v**_**i**_ is the corresponding eigenvector with *k* components, and the eigenvectors are orthonormal. Let *μ*_(−*i*)_ = *μ*_*i*_ for all *i*, then *Vn* is invariant to centring around *μ*_*i*_; thus, the distributions of *Vn* when **z** = **w**^1/2^**y** and when **z** = **w**^1/2^(**y** − **μ**) are equivalent. In the latter case, for ***z*** = (*z*_1_, *z*_2_, *z*_3_, …,*z*_*k*_) each *z*_*i*_ ~ *N*(0, 1) and is independent.

Because **v**_**i**_ are orthonormal, they form an alternative basis that is a rotation of the usual standard basis in Euclidean space. Supposing $v_{i}^{T}=\left(v_{i1},v_{i2},\ldots {,v}_{\mathrm{ik}}\right)$, then $v_{i}^{T}z=v_{i1}z_{1}+v_{i2}z_{2}+\ldots {+v}_{\mathrm{ik}}z_{k}$. Thus, $v_{i}^{T}z$ is a linear combination of standard normal variables and, as a result, has a normal distribution. Furthermore, the expectation, $E\left(v_{i}^{T}z\right)=0$ because *E*(*z*_*i*_) = 0 and the variance, $\mathrm{var}\left(v_{i}^{T}z\right)=1$, because var(*z*_*i*_) = 1 and $\|v_{i}^{T}\|=1$. Hence, $v_{i}^{T}z\;$~*N* (0, 1)_,_ and thus ${\left(v_{i}^{T}z\right)}^{2}$ **~** $\chi_{1}^{2}$. So we have

(A2) $$\mathrm{Vn}∼\sum_{i=1}^{k}\lambda_{i}\;\chi_{1}^{2}$$

and therefore *Vn* has a distribution which is a linear combination of *χ*^2^ variables of degree 1 where the coefficients are the eigenvalues of **B**. This is an asymptotic distribution because $\sigma_{i}^{2}$ and τ^2^ are estimated from the sample data. We note the property that the entries of each row of **A** sum to zero persists in **A**^**T**^**w**^*^**A** **.** Furthermore, because this matrix is symmetrical, the entries of each column also sum to zero. **B** = **w**^−1/2^**A**^**T**^**w**^*^**Aw**^−1/2^ is symmetrical, and if we multiply each row *i*, by a factor (*w*_1_*w*_2_. . *w*_*i* − 1_*w*_*i* + 1_ .  . *w*_*k*_)^−1/2^ where *w*_*j*_ is the *jt*h element on the diagonal of **w**, the column entries also sum to zero; thus, **B** has rank = *k* − 1. Because *Vn* is non‐negative, **B** is positive semi‐definite and has *k* − 1 positive eigenvalues, where the *k*th eigenvalue, *λ*_*k*_ = 0. Furthermore, when the non‐zero eigenvalues all equal one, *Vn* has a *χ*^2^ distribution of *k* − 1 degrees of freedom.

(ii) *Tailored meta‐regression model (2)*

To validate the summary estimate $X_{i}{\hat{\beta }}_{\left(-i\right)}$ from the meta‐regression model in (2), it is again of interest to formulate *Vn* in matrix form. Note **X**_*i*_ is the row vector for the *ith* study with 1 as the first element and each of the *p* − 1 covariates as the other elements. Let $\theta_{\left(-i\right)}={\left(X_{\left(-i\right)}^{T}w_{\left(-i\right)}^{*}X_{\left(-i\right)}\right)}^{-1}X_{\left(-i\right)}^{T}w_{\left(-i\right)}^{*}$, where **X**_(−*i*)_ is the matrix of row vectors **X**_*j*_ with the *i*th study excluded. Thus, **θ**_(−**i**)_ is a *p × (k* − 1*)* matrix for *p* − 1 covariates, and ${\hat{\beta }}_{\left(-i\right)}=\theta_{\left(-i\right)}y_{\left(-i\right)}$. Suppose we partition **θ**_(−**i**)_ into the first *i* − 1 columns, append an *i*th column of zeros, then re‐append the remaining *k* − *i* columns to produce ${\hat{M}}_{\left(-i\right)}$ a *p × k* matrix.

We define the matrix **A** as having elements

(A3) Aij=1ifi=j−XiM^j−iifi≠j

where ${\hat{M}}_{j\left(-i\right)}$ is the *jth* column of ${\hat{M}}_{\left(-i\right)}$ of length *p*. As a result, *Vn* may be written in the quadratic form

$$\mathrm{Vn}=y^{T}A^{T}w^{*}\mathrm{Ay}$$

where ***w***^*^ is defined as previously and **A**^**T**^**w**^*^**A** is symmetrical. By similar arguments to those made in Section 3.3(i), we may again define **z** = **w**^1/2^**y** and write **B** = **w**^−1/2^**A**^**T**^**w**^*^**Aw**^−1/2^ in order to deduce that

(A4) $$\mathrm{Vn}∼\sum_{i=1}^{k}\lambda_{i}\;\chi_{1}^{2}$$

The matrix **θ**_(−**i**)_ has rank *p* because **X**_(−***i***)_ has rank *p*. The *k*‐vector **Ay** is analogous to (**I** − **H**)**y** the vector of residuals **(** $y_{i}-\hat{y}$ **)** where **H** is the hat matrix. **Ay** represents the vector of predictive residuals $\left(y_{i}-{\hat{y}}_{\left(-i\right)}\right)$ across all **X**_***i***_ and *y*_*i*_. Thus, **A** spans the same sub‐space as **I** − **H** and has rank *k‐p.*

The rank of **w**^*^**Aw**^−1/2^ is the same as **A** because both **w**^*^ and **w**^−1/2^ are diagonal and the transpose, **A**^**T**^, also has the same rank as **A**. Thus, **B** = **w**^−1/2^**A**^**T**^**w**^*^**Aw**^−1/2^ has rank *k‐p.* Again, **B** is positive semi‐definite and has *k‐p* positive eigenvalues, where *λ*_*k* − *p* + 1_ =  …  = *λ*_*k*_ = 0 for *p < k*.

## Appendix 2 R source code to estimate Vn for Berkey et al.

### 2.2

This estimates Vn for meta‐analysis and tailored meta‐regression with 1 covariate

library(metafor)

library(CompQuadForm)

c&lt;-read.csv("Berkey file.csv")

total&lt;-data.frame(total1P=numeric(), exact_p1=numeric(),total2P=numeric(),exact_p2=numeric())

n&lt;-nrow(c)

A1&lt;-A2&lt;-0

wt1&lt;-wt2&lt;-0

B1&lt;-B2&lt;-0

Vn1&lt;-Vn2&lt;-0

for (i in 1:n)

{

a &lt;- c[i,]

b &lt;- c[-i,]

##################################################################################

## Study RR, yi and variance

RRi&lt;-(a$tpos/(a$tpos+a$tneg))/(a$cpos/(a$cpos+a$cneg))

yi&lt;- log(RRi)

varyi&lt;-1/(a$tpos) + 1/(a$cpos) - 1/(a$tpos+a$tneg) - 1/(a$cpos+a$cneg)

##################################################################################

# Estimate the y(i)=RR(i) when no covariates

estim1 &lt;- rma(ai=b$tpos, n1i=b$tpos+b$tneg,ci=b$cpos, n2i=b$cpos+b$cneg, data=b, add=1/2,to="only0",method ="REML", measure="RR", control=list(stepadj=.5))

PredYib &lt;- predict(estim1, addx=TRUE)

Yib&lt;- PredYib$pred

varYib1&lt;- (PredYib$se)^2

Vn1[i]&lt;-((yi-Yib)^2)/(varyi+varYib1)

t1&lt;-estim1$tau2

tau1&lt;-c(rep(t1,n-1))

varY1&lt;-1/(b$tpos) + 1/(b$cpos) - 1/(b$tpos+b$tneg) - 1/(b$cpos+b$cneg)

weight1&lt;-1/(tau1+varY1)

W1&lt;-sum(weight1)

weight1&lt;--weight1/W1

wt1i&lt;-1/((1/W1)+varyi)

if (i==1) x&lt;-c(1,weight1)

if (i>1 &amp; i&lt;n)

{

x1&lt;-weight1[1:(i-1)]

x2&lt;-weight1[i:(n-1)]

x&lt;-c(x1,1,x2)

}

if (i==n) x&lt;-c(weight1,1)

wt1&lt;-c(wt1,wt1i)

A1&lt;-c(A1,x)

########################################################################

# Estimate the y(i)=RR(i) when 1 covariate (LATITUDE)

estim2 &lt;- rma(ai=b$tpos, n1i=b$tpos+b$tneg,ci=b$cpos, n2i=b$cpos+b$cneg, data=b, add=1/2,to="only0",mods=~b$Lat, method ="REML",measure="RR", control=list(stepadj=.5))

PredYib2 &lt;- predict(estim2,newmods=a$Lat, addx=TRUE)

Yib2&lt;- PredYib2$pred

varYib2&lt;- (PredYib2$se)^2

Vn2[i]&lt;-((yi-Yib2)^2)/(varyi+varYib2)

t2&lt;-estim2$tau2 # Between study variance

tau2&lt;-c(rep(t2,n-1)) #Vector of n-1 between study variances

weight2&lt;-1/(tau2+varY1) #within-study variances already estimated above

w&lt;-diag(weight2)

X2&lt;-matrix(c(rep(1,n-1),b$Lat),n-1,byrow=F)

xi2&lt;-matrix(c(1,a$Lat),1,byrow=F)

inv&lt;- solve(t(X2)%*%w%*%X2)

temp&lt;-xi2%*%inv%*%t(X2)%*%w

temp&lt;--1*temp

if (i==1) dum&lt;-matrix(c(1,temp),1,byrow=F)

if (i>1 &amp; i&lt;n)

{

temp1&lt;-matrix(temp[1:(i-1)],1,byrow=F)

temp2&lt;-matrix(temp[i:(n-1)],1,byrow=F)

dum&lt;-matrix(c(temp1,1,temp2),1,byrow=F)

}

if (i==n) dum&lt;-matrix(c(temp,1),1,byrow=F)

if (i==1) A2&lt;-dum

else A2&lt;-rbind(A2,dum)

wt2i&lt;-1/(varYib2+varyi)

wt2&lt;-c(wt2,wt2i)

##################################################################################

}

## Distribution of Vn

## Case - no covariates

SEc&lt;-(1/(c$tpos) + 1/(c$cpos) - 1/(c$tpos+c$tneg) - 1/(c$cpos+c$cneg))^0.5

YiF&lt;-log((c$tpos/(c$tpos+c$tneg))/(c$cpos/(c$cpos+c$cneg)))

y&lt;-matrix(YiF,nrow=n,ncol=1)

wt1&lt;-wt1[-1]

wt1&lt;-diag(wt1)

zwt&lt;-diag(SEc)

A1&lt;-A1[-1]

A1&lt;-matrix(A1,nrow=n,ncol=n,byrow=TRUE)#Note byrow if FALSE by default

B1&lt;-t(zwt)%*%t(A1)%*%wt1%*%A1%*%zwt

e_values1&lt;-eigen(B1,symmetric=TRUE)

eval1&lt;-e_values1$values

eval1&lt;-eval1[-n]

total1&lt;-sum(Vn1)

## To estimate the distribution of Vn

chis1&lt;-c(rep(1,n-1))

cents1&lt;-c(rep(0,n-1))

exact1&lt;-farebrother(total1,eval1,chis1,cents1) #Vn, vector of e-values, vector of DOF for chi, vector of zeros for centrally distributed chi

exact_p1&lt;-exact1$res

##############################################

## Distribution of Vn

## Case - 1 covariate (LATITUDE)

wt2&lt;-wt2[-1]

wt2&lt;-diag(wt2)

temp4&lt;-t(A2)%*%wt2%*%A2

B2&lt;-t(zwt)%*%temp4%*%zwt

total2&lt;-sum(Vn2)

e_values2&lt;-eigen(B2,symmetric=TRUE)

eval2&lt;-e_values2$values

eval2&lt;-eval2[-((n-1):n)]

## To estimate the distribution of Vn

chis2&lt;-c(rep(1,n-2)) #

cents2&lt;-c(rep(0,n-2))

exact2&lt;-farebrother(total2,eval2,chis2,cents2) #Vn, vector of e-values, vector of dof for chi, vector of zeros for centrally distributed chi

exact_p2&lt;-exact2$res

total&lt;-data.frame(total1,exact_p1,total2,exact_p2)

total
