# Supplementary material for: Measuring the statistical validity of summary meta‐analysis and meta‐regression results for use in clinical practice
Source: Stat Med. 2017 Jun 15;36(21):3283–301. doi: 10.1002/sim.7372 (PMC5575530; doi:10.1002/sim.7372)
Supplement: Supplementary file 1 — Data S1. Supporting info item [file SIM-36-3283-s001.docx]

**Online Appendix**

**Citations from sample of systematic reviews**

1. Fraquelli M, Casazza G, Conte D, Colli A. Non-steroid anti-inflammatory drugs for biliary colic.Cochrane Database of Systematic Reviews 2016, Issue 9. Art. No.: CD006390. DOI: 10.1002/14651858.CD006390.pub2.
2. Martineau AR, Cates CJ, Urashima M, Jensen M, Griffiths AP, Nurmatov U, Sheikh A, Griffiths CJ.Vitamin D for the management of asthma. Cochrane Database of Systematic Reviews 2016, Issue 9. Art. No.: CD011511. DOI: 10.1002/14651858.CD011511.pub2.
3. Clarke MJ, Broderick C, Hopewell S, Juszczak E, Eisinga A. Compression stockings for preventing deep vein thrombosis in airline passengers. Cochrane Database of Systematic Reviews 2016, Issue 9. Art. No.: CD004002. DOI: 10.1002/14651858.CD004002.pub3.
4. Wong WT, Lai VKW, Chee YE, Lee A. Fast-track cardiac care for adult cardiac surgical patients.Cochrane Database of Systematic Reviews 2016, Issue 9. Art. No.: CD003587. DOI: 10.1002/14651858.CD003587.pub3.
5. Shepperd S, Iliffe S, Doll HA, Clarke MJ, Kalra L, Wilson AD, Gonçalves-Bradley DC. Admission avoidance hospital at home. Cochrane Database of Systematic Reviews 2016, Issue 9. Art. No.: CD007491. DOI: 10.1002/14651858.CD007491.pub2.
6. Greenough A, Rossor TE, Sundaresan A, Murthy V, Milner AD. Synchronized mechanical ventilation for respiratory support in newborn infants. Cochrane Database of Systematic Reviews 2016, Issue 9. Art. No.: CD000456. DOI: 10.1002/14651858.CD000456.pub5.
7. Prabhakar H, Singh GP, Mahajan C, Kapoor I, Kalaivani M, Anand V. Intravenous versus inhalational techniques for rapid emergence from anaesthesia in patients undergoing brain tumour surgery. Cochrane Database of Systematic Reviews 2016, Issue 9. Art. No.: CD010467. DOI: 10.1002/14651858.CD010467.pub2.
8. Sng BL, Siddiqui FJ, Leong WL, Assam PN, Chan ESY, Tan KH, Sia AT. Hyperbaric versus isobaric bupivacaine for spinal anaesthesia for caesarean section. Cochrane Database of Systematic Reviews 2016, Issue 9. Art. No.: CD005143. DOI: 10.1002/14651858.CD005143.pub3.
9. Chin KJ, Cubillos JE, Alakkad H. Single, double or multiple-injection techniques for non-ultrasound guided axillary brachial plexus block in adults undergoing surgery of the lower arm.Cochrane Database of Systematic Reviews 2016, Issue 9. Art. No.: CD003842. DOI: 10.1002/14651858.CD003842.pub5.
10. van Driel ML, De Sutter AIM, Habraken H, Thorning S, Christiaens T. Different antibiotic treatments for group A streptococcal pharyngitis. Cochrane Database of Systematic Reviews2016, Issue 9. Art. No.: CD004406. DOI: 10.1002/14651858.CD004406.pub4.
11. Kakkos SK, Caprini JA, Geroulakos G, Nicolaides AN, Stansby G, Reddy DJ, Ntouvas I. Combined intermittent pneumatic leg compression and pharmacological prophylaxis for prevention of venous thromboembolism. Cochrane Database of Systematic Reviews 2016, Issue 9. Art. No.: CD005258. DOI: 10.1002/14651858.CD005258.pub3.
12. Leeflang MM, Debets-Ossenkopp YJ, Visser CE, Scholten RJPM, Hooft L, Bijlmer HA, Reitsma JB, Bossuyt PMM, Vandenbroucke-Grauls CM. Galactomannan detection for invasive aspergillosis in immunocompromized patients. *Cochrane Database of Systematic Reviews 2008, Issue 4. Art. No.: CD007394. DOI: 10.1002/14651858.CD007394*
13. Wilkinson P, Izmeth Z. Continuation and maintenance treatments for depression in older people. Cochrane Database of Systematic Reviews 2016, Issue 9. Art. No.: CD006727. DOI: 10.1002/14651858.CD006727.pub3.
14. Bighelli I, Trespidi C, Castellazzi M, Cipriani A, Furukawa TA, Girlanda F, Guaiana G, Koesters M, Barbui C. Antidepressants and benzodiazepines for panic disorder in adults. Cochrane Database of Systematic Reviews 2016, Issue 9. Art. No.: CD011567. DOI: 10.1002/14651858.CD011567.pub2.
15. Theron G, Peter J, Richardson M, Warren R, Dheda K, Steingart KR. GenoType^®^ MTBDRsl assay for resistance to second-line anti-tuberculosis drugs. Cochrane Database of Systematic Reviews2016, Issue 9. Art. No.: CD010705. DOI: 10.1002/14651858.CD010705.pub3.
16. Berkey CS, Hoaglin DC, Mosteller F, Colditz GA. A random-effects regression model for meta-analysis. *Stat Med* 1995;14:395-411.
